# Ethics views on animal science and animal production

**DOI:** 10.1093/af/vfz049

**Published:** 2020-01-10

**Authors:** Bart Gremmen

**Affiliations:** Department of Philosophy, Wageningen University, Wageningen, The Netherlands

In the last century, animal science has had more than a fair share in securing agriculture’s dominant task of feeding the human population. From an ethical perspective, this is clearly very positive, but it does not absolve animal scientists from critical, ethical examination of the consequences of their work.

To earn the public’s ongoing support, animal scientists must be trusted to align with the highest ethical values. Animal scientists need to do more to address the broader ethical issues that are of increasing concern to the public. Animal scientists need to be proactive and propose an ethical agenda about innovations. What do we mean by ethical reflection in the field of animal sciences? Ethics may be studied from several disciplinary backgrounds: law, theology, psychology, philosophy, or social science. In this special issue (Figure 1), ethics is studied mainly from a philosophical background and defined as the critical, systematic reflection on implicit and explicit moral assumptions of animal scientists. The aim is to offer a series of papers dealing with different proactive and constructive ways to deal with ethical conflicts. We also want the different ethical views to contribute to responsible policies and practices by enriching the reflection of societal groups, policymakers, professionals, and NGOs with questions from the perspective of animal ethics.

**Figure 1.**
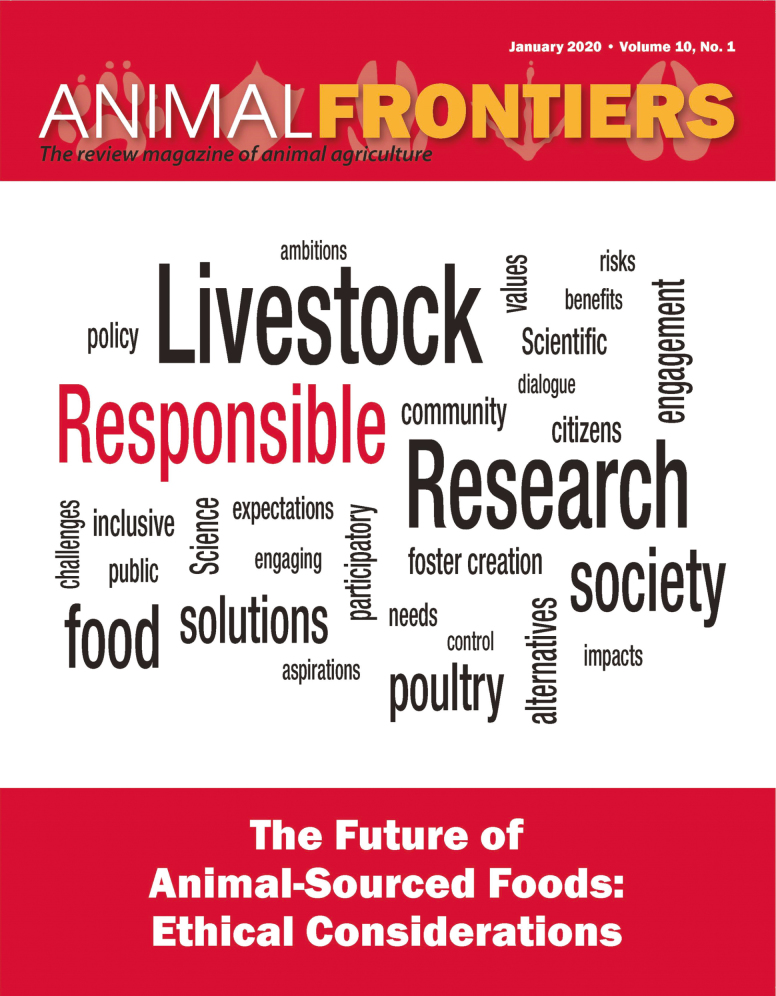
The future of animal-sourced foods: ethical considerations.

In the past, many papers have been published about the ethics of animal science and animal production. This has led to a range of different themes and views. In this special issue, we will organize a number of these views in two sections. The first section provides an overview of different views on animals and animal production systems: views on animals in different religions (Caruara, 2020), three different views on the ethics of animal production systems (Gremmen, 2020; Figure 2), and different ethical views on factory farms (Thompson, 2020). The second section focuses on three core themes from the literature on the ethics of animal science and animal production: meat (Francione, 2020; Pulina, 2020), modern biotechnology (Bovenkerk, 2020; Shriver, 2020), and Precision Livestock Farming (Werkheiser, 2020).

**Figure 2.**
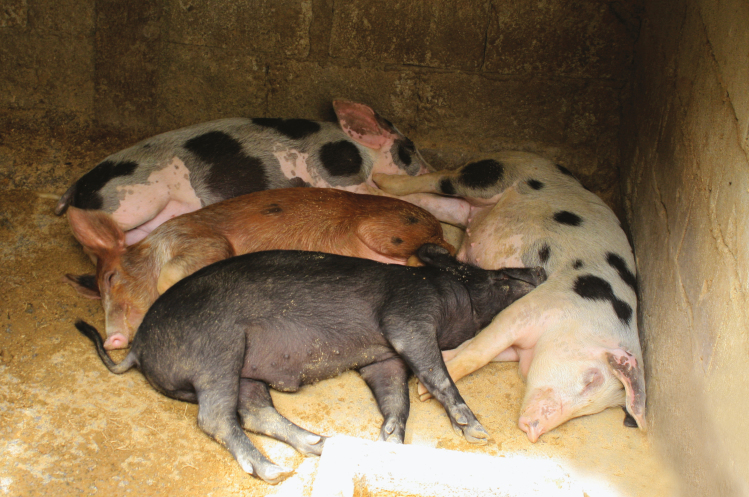
Sleeping native pigs raised in an organic production system in the Phillipines.

Caruara (2020) provides a chronological overview of the ethical views on animals in different religions and explains that religions, in spite of their differences, converge on some fundamental points, some of which concern our responsibility toward animals. Human superiority over all other creatures is to be understood in terms of caring for creation. Moral questions concerning our treatment of fellow humans are linked to those concerning our treatment of animals. Animal care is an obligation, both moral and religious.

Gremmen (2020) argues that an ethics of animal production “systems” consists of a Moral Operating System, which consists of an “internal” professional “care” ethics, an “external animal” ethics, and an “emergent” ethics in life sciences enabling change by responsible innovation. A Moral Operating System will help scientists, stakeholders, and policymakers to understand, evaluate, and monitor the integration of ethical aspects of agricultural systems.

Thompson (2020) focuses on the ethical aspects of factory farms. He states that philosophers have neglected the relationships that establish duties to farmed animals, especially in factory farms. Many philosophers apparently assume that the conditions in industrial facilities are so horrible that the very idea of discussing obligations to them is vitiated by the unredeemable nature of the circumstances in which they live. Even when widely read texts accurately describe welfare deficits, they present a picture which is misleading both as to the extent of these problems and to difficulty of making changes in response to them. Frequently cited welfare problems in factory farms support a case for reform, but it is difficult to see how it would support the claim that what is done to farmed animals is equivalent to torture. Philosophical analysis could actually help improve the quality of life for animals living on factory farms.

The second section of this special issue starts with the paper of Francione (2020) who argues against the use of meat, while Pulina (2020) is defending the use of meat. According to Francione (2020), it is absurd that some animal rights campaigners maintain that we should allow animals the same legal rights enjoyed by humans. A sensible and coherent theory of animal rights should focus on just “one” right for animals—the right not to be treated as the property of humans. Recognizing animal rights really means accepting that we have a duty not to treat sentient nonhumans as resources. Pulina (2020) defends the opposite position. In his view, meat consumption is morally justified and animals are not carriers of rights. People have specific obligations toward animals. The first obligation is to respect animals and to guarantee their well-being, which must represent the main concern of a breeder. This paper chose an operative position: what does good or evil mean for animals and how should this be interpreted for positive purposes, including the production of food for humans. Because, before anything else, ethics pursues the aim of improving the lives of Men and other living beings while respecting the fundamental natural and cultural principles that govern the biosphere and human societies.

The second theme starts with Shriver’s (2020) ethical analysis of the relation between modern biotechnology and welfare. He develops arguments for the claim that a Principle for the Conservation of Welfare should be adopted to ensure that the genetic modification of livestock does not result in unnecessary suffering. Failing do so would both be morally wrong and likely to result in a serious undermining of public trust in food producers. This principle needs to be enshrined in legislation or regulation in order to be effective and assuming that the principle will be followed via “self-regulation” would be both morally wrong and likely to permanently damage trust in food producers.

In her paper about genetic modification of animals, Bovenkerk (2020) aims to go “beyond” welfare arguments. She starts with the observation that many people still have moral problems with modified animals, whether or not they experience welfare problems. The arguments “beyond welfare” appear to be part of broader conceptions of the “good life” and of how to be a good person. There is less agreement on the arguments beyond welfare, which rely on people’s comprehensive notions of the good life, about which people disagree fundamentally. By only taking rule-ethical principles seriously, many important values and meanings that people attach to life and to the world around them are disregarded. We do not blindly employ our technologies on animals, but we should once in a while step back and reflect on what modifications mean for our relationship to animals and nature and on what kind of world we want to live in.

In his paper about Precision Livestock Farming, Werkheiser (2020) states that there is a lot of pressure from various quarters for traditional farms to scale up, yet doing so brings with it a host of problems around animal husbandry. We have also seen that Precision Livestock Farming is a promising solution, but one with a host of concerns that are currently very underexamined and underdiscussed, particularly outside academia. None of these concerns are so damning that Precision Livestock Farming should not be pursued. However, they all require careful negotiation and forethought and the incorporation of the perspectives of many stakeholders.

In summary, all papers in this issue describe and analyze many interesting views on the ethics of animal science and animal production. The Ethics Working Group of the European Federation of Animal Science aims to provide a focal point for those who have a professional interest in the ethical issues involved in animal science. It is an interdisciplinary, cross-cultural and non-partisan network of international experts, scientists, and professionals with special interest in the ethical issues of physiology, nutrition, breeding and genetics, husbandry and management, welfare and animal health, milk, meat and fiber production, etc. Membership of the Ethics Working Group is voluntary, based on scientific interest.

## About the Author

**Prof. Dr. Bart Gremmen** is based at the Philosophy chair Group, known for applying ethical theory to life science domains with a strong focus on plant, animal, and environmental ethics. His membership of the Plant Breeding group (2004-) and the Adaptation physiology group (2010-) and his collaboration with different Wageningen University and Research groups (environmental science; technology innovation; strategic communication) ensures a multidisciplinary approach. Prof. Gremmen is a member of the environmental and ethics groups of the Netherlands Research School of Philosophy, and he has coordinated three large Nederlandse Organisatie voor Wetenschappelijk Onderzoek genomics programs. He is member of the management team of the 4Technical Universities Centre for Ethics of Technology and has established a strong international network of environmental and applied ethicists. Prof. Gremmen is president of the International Society for the Hermeneutics of science. He is also chair of the Ethics working group of the European Association of Animal Scientists.
